# Supplementary material for: Identification of photoperiod-induced specific miRNAs in the adrenal glands of Sunite sheep (Ovis aries)
Source: Front Vet Sci. 2022 Jul 22;9:888207. doi: 10.3389/fvets.2022.888207 (PMC9354845; doi:10.3389/fvets.2022.888207)
Supplement: Supplementary file 1 [file Data_Sheet_1.ZIP › Supplementary file/miRDeep2 file.zip!/miRDeep2 file/Novel_Structure/SPLP42A1/Novel_170.pdf]

| | | |
|---|---|---|
| Provisional ID | : | NC_019461.2_9799 |
| Score total | : | 1 |
| Score for star read(s) | : | -1.3 |
| Score for read counts | : | -0.8 |
| Score for mfe | : | 1.6 |
| Score for randfold | : | 1.6 |
| Score for cons. seed | : | |
| Total read count | : | 10 |
| Mature read count | : | 10 |
| Loop read count | : | 0 |
| Star read count | : | 0 |

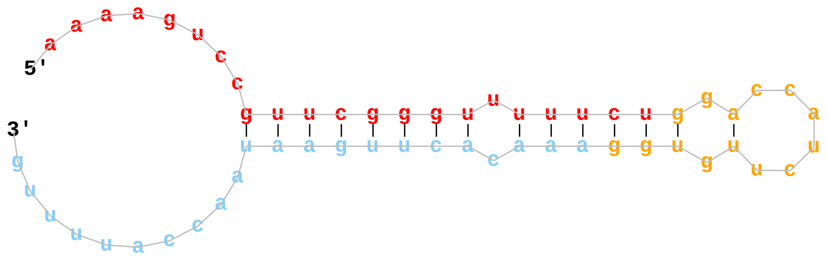


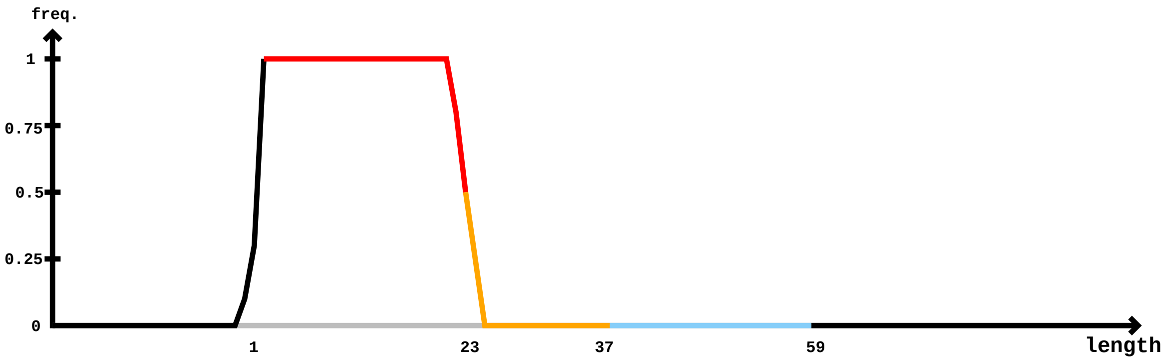


```
                    Mature                          Star
5'- gggauguauuagguuggccaaaaaguccguucggguuuuucuggaccaucuuguggaaacacuugaauaaccauuuuggccaaccccguacuuuguaguacauacacacgu   -3'    exp
    .(..(((((..((((((((((((.(...((((((((.((((((.(......).)))))).))))))))...).))))))))))))..(((((....))))))))))..)..    reads    mm    sample
    ..................caaaaaguUcguucggguuuuu.......................................................................    1        1     seq
    ...................aaaaaguUcguucggguuuuuc......................................................................    2        1     seq
    ....................aaaaguccguucggguuuuu.......................................................................    1        0     seq
    ....................aaaaguccguucggguuuuuc......................................................................    1        0     seq
    ....................aaaaguUcguucggguuuuucu.....................................................................    5        1     seq
```
